# Is Every Woman a Nurse?

**Published:** 1918-11-23

**Authors:** 


*To the Editor of* THE HOSPITAL.

### "Is Every Woman a Nurse?"

*To the Editor of* THE HOSPITAL.

SIR,—Your correspondent "Liverpool" challenges "Ierne's" statement, quoted from Florence Nightingale, that "every woman is a nurse," and points out, from the standpoint of matrons of training-schools, that it is not true. May I just say that the matrons of training-schools are not necessarily in a position to judge? The conditions of work in many training-schools are so severe that the nursing impulse may be crushed. When a young girl joins as a probationer she may be very enthusiastic about nursing, but when she has to be up at six o'clock in the morning and has to work until ten at night with very little time off, day by day and week by week, and conform to rigid rules of discipline to which she was never previously subjected, she may develop a loathing for hospital life. This often happens, but it is a mistake to suppose that nursing the sick is responsible for the revulsion of feeling. It is the slavery that makes the life unbearable, which I think is "Ierne's" point.

Women sometimes revolt against marriage. Why? Because if the income is only large enough to support one, and a family have to be reared on it, life becomes one long grey misery. For vivid examples see the dwellers in the slums. Give every woman her choice of being, say, a successful spinster, or marrying the man of her choice who possesses a competence, what answer will Nature inspire her to offer? Every man who wishes for an outdoor life might not care to be a policeman. I am disposed to think that if nursing conditions are revolutionised there will be no lack of top-notch candidates.

CHRYSANTHEMUM.

